# Supplementary figures and images for: The oldest record of the Steller sea lion Eumetopias jubatus (Schreber, 1776) from the early Pleistocene of the North Pacific
Source: PeerJ. 2020 Aug 27;8:e9709. doi: 10.7717/peerj.9709 (PMC7456534; doi:10.7717/peerj.9709)

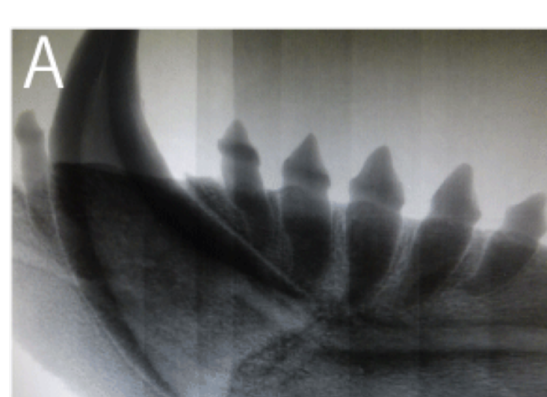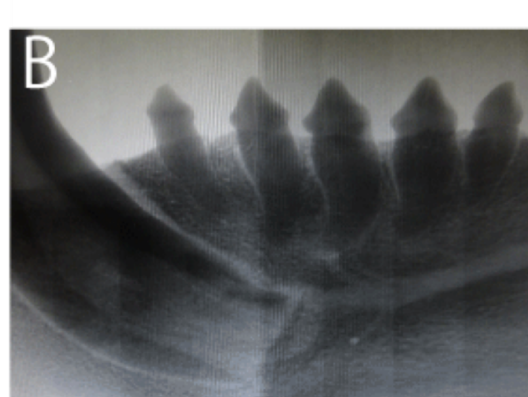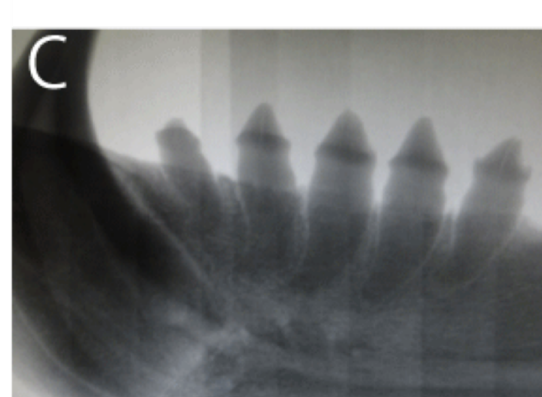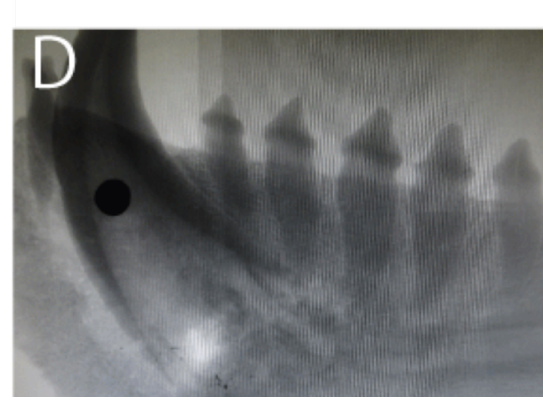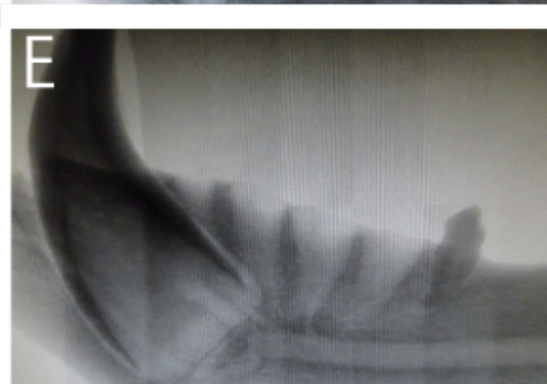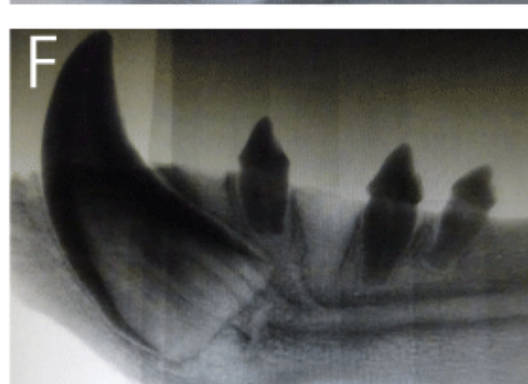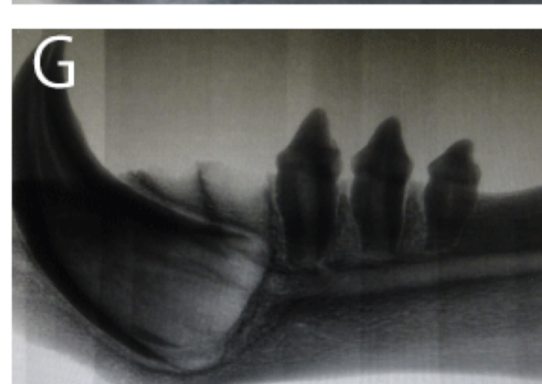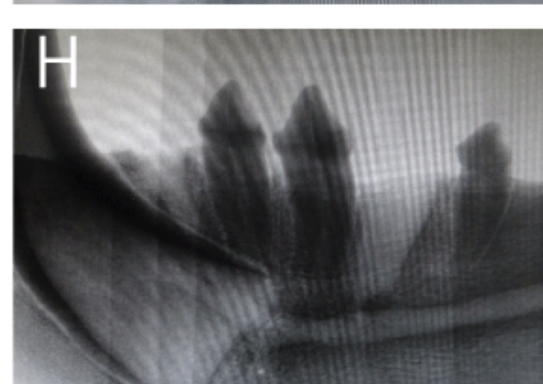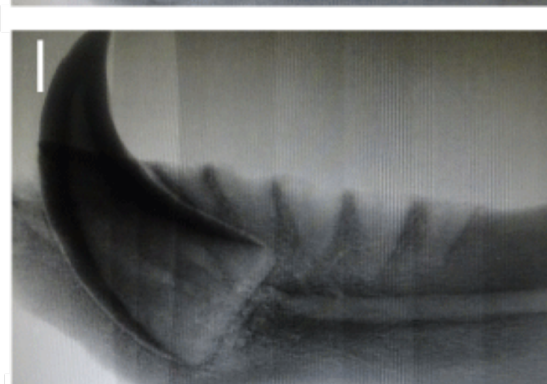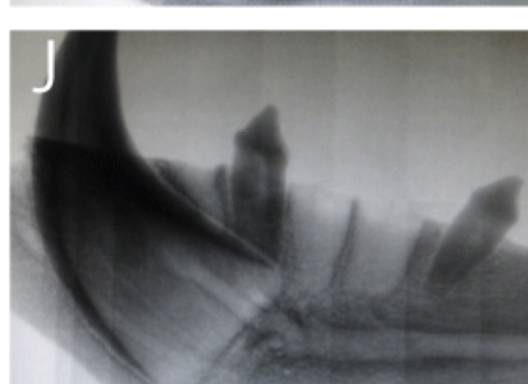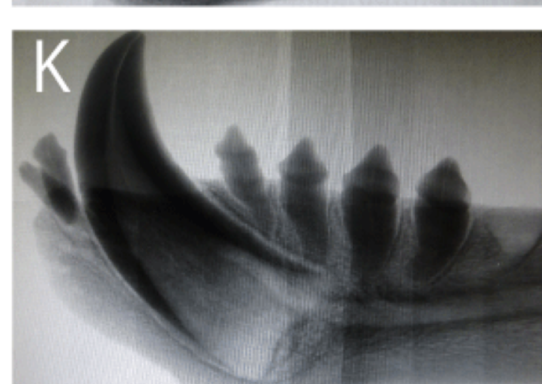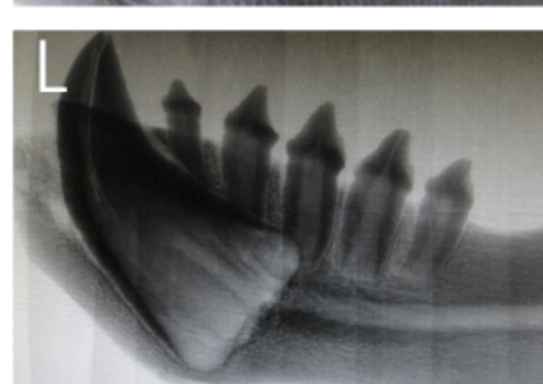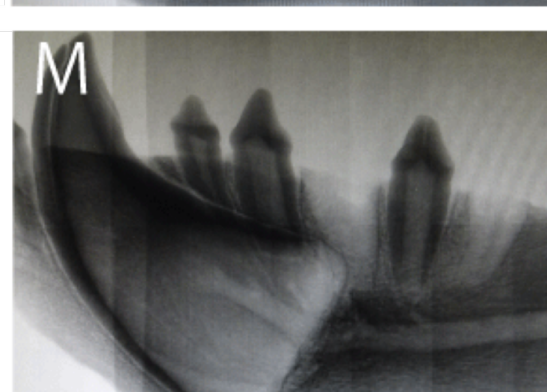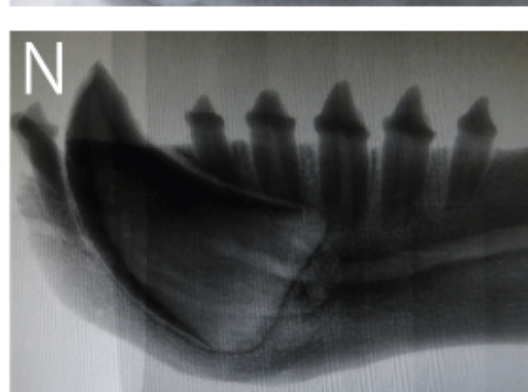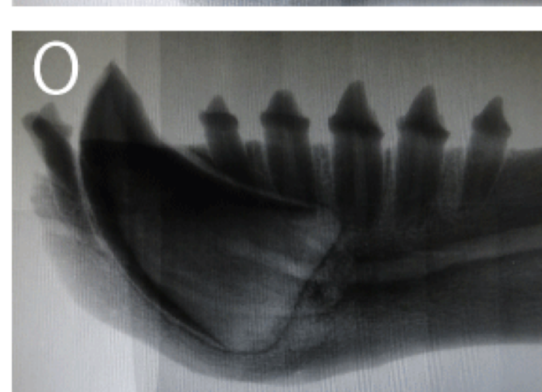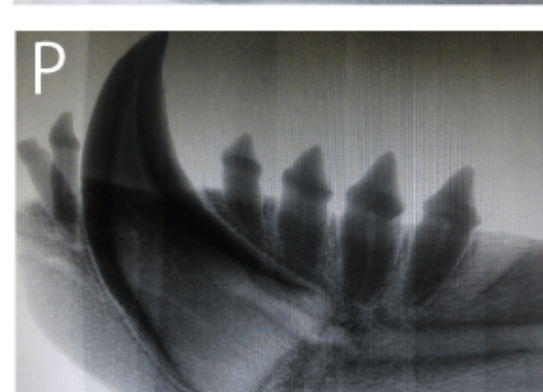

Supplement: Supplemental Information 1 — (A) NMNS-KK 14 (B) NMNS-KK 15 (C) NMNS-KK 23 (D) NMNS-KK 42 (E) NMNS-KK 51 (F) NMNS-KK 63 (G) NMNS-KK 65 (H) NMNS-KK 69 (I) NMNS-KK 71 (J) NMNS-KK 73 (K) NMNS-KK 105 (L)NMNS-KK 145 (M) NMNS-KK 164 (N)NMNS-KK 167 (O)NMNS-KK 169 (P) NMNS-KK 192. Photo credit: Chisako Sakata. [file peerj-08-9709-s001.pdf]
