# Supplementary material for: The oldest record of the Steller sea lion Eumetopias jubatus (Schreber, 1776) from the early Pleistocene of the North Pacific
Source: PeerJ. 2020 Aug 27;8:e9709. doi: 10.7717/peerj.9709 (PMC7456534; doi:10.7717/peerj.9709)
Supplement: Supplemental Information 2 [file peerj-08-9709-s002.docx]

Plate 1

Specimens of male *Eumetopias jubatus* measured in this study. The upper row shows lateral aspect, the middle row shows medial aspect, and the lower row shows dorsal aspect, respectively. The scale shows 10 cm.

1：NMNS-KK 14

2：NMNS-KK 15

3：NMNS-KK 23

4：NMNS-KK 51

5：NMNS-KK 63

6：NMNS-KK 69


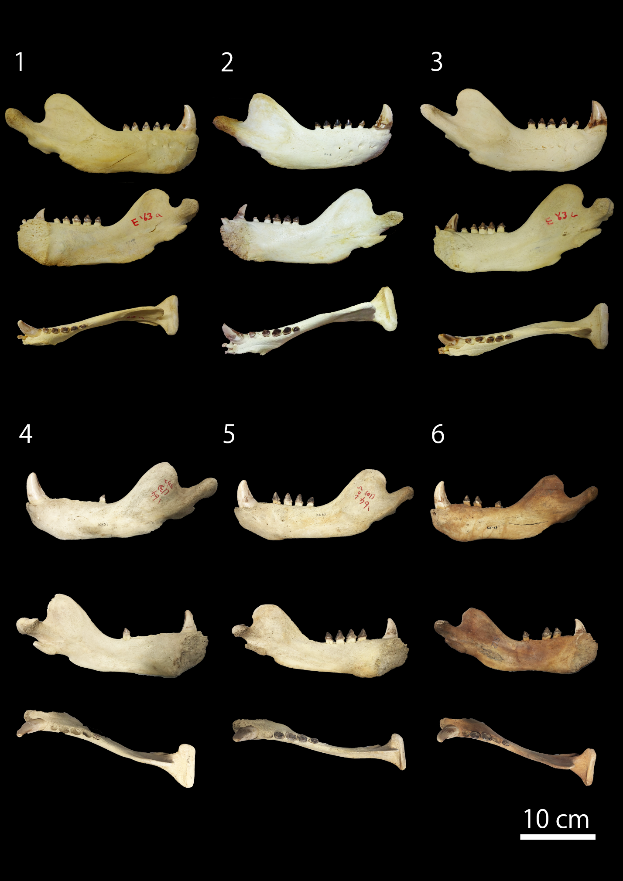
Plate 1

Plate 2

Specimens of male *Eumetopias jubatus* measured in this study. The upper row shows lateral aspect, the middle row shows medial aspect, and the lower row shows dorsal aspect, respectively. The scale shows 10 cm.

1：NMNS-KK 73

2：NMNS-KK 167

3：NMNS-KK 169

4：NMNS-KK 192

5：NMNS-M 5627

6：NMNS-M 28387


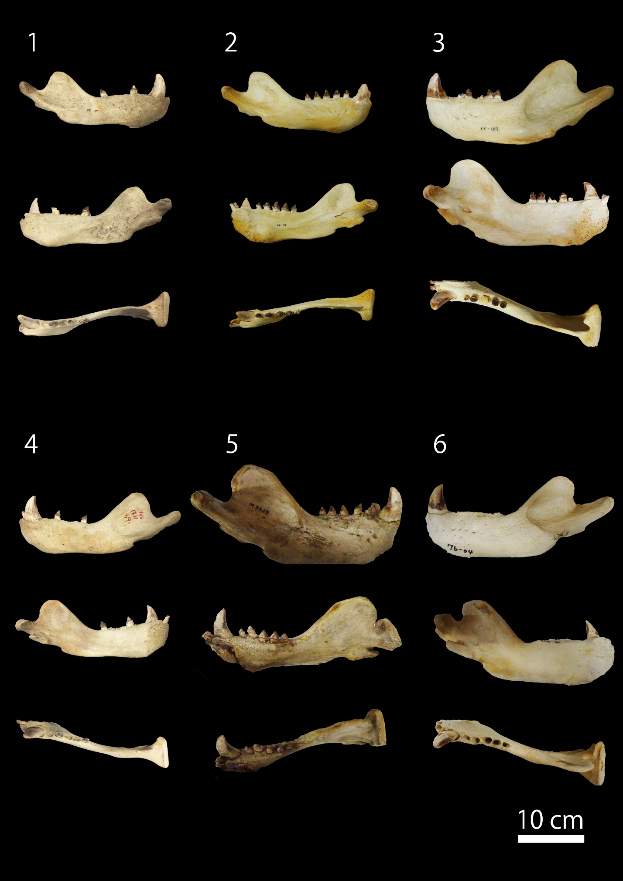
Plate 2

Plate 3

Specimens of female *Eumetopias jubatus* measured in this study. The upper row shows lateral aspect, the middle row shows medial aspect, and the lower row shows dorsal aspect, respectively. The scale shows 10 cm.

1：NMNS-KK 11

2：NMNS-KK 53

3：NMNS-KK 54

4：NMNS-KK 55

5：NMNS-KK 56

6：NMNS-KK 139


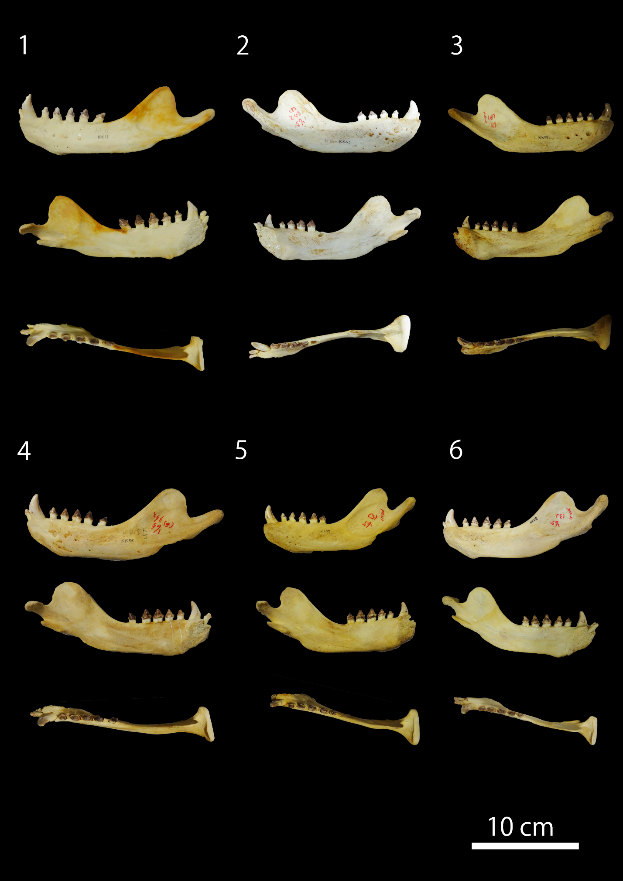
Plate3

Plate 4

Specimens of female *Eumetopias jubatus* measured in this study. The upper row shows lateral aspect, the middle row shows medial aspect, and the lower row shows dorsal aspect, respectively. The scale shows 10 cm.

1：NMNS-KK 146

2：NMNS-KK 154

3：NMNS-KK 158

4：NMNS-KK 165

5：NMNS-KK 166

6：NMNS-M 17123


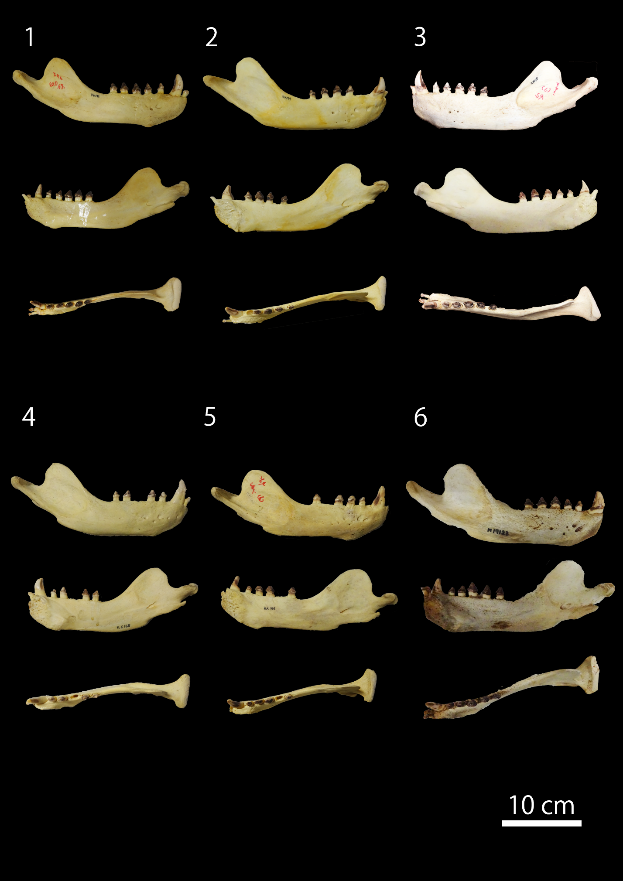
Plate4

Plate 5

Specimens of *Callorhinus ursinus* measured in this study. The upper row shows lateral aspect, the middle row shows medial aspect, and the lower row shows dorsal aspect, respectively. The scale shows 10 cm.

1：male, NMNS-M 2454

2：male, NMNS-M 17140

3：male, NMNS-M 46874

4：female, NMNS-KK 05

5：female, NMNS-KK 08

6：female, NMNS-KK 10

7：female, NMNS-KK 22


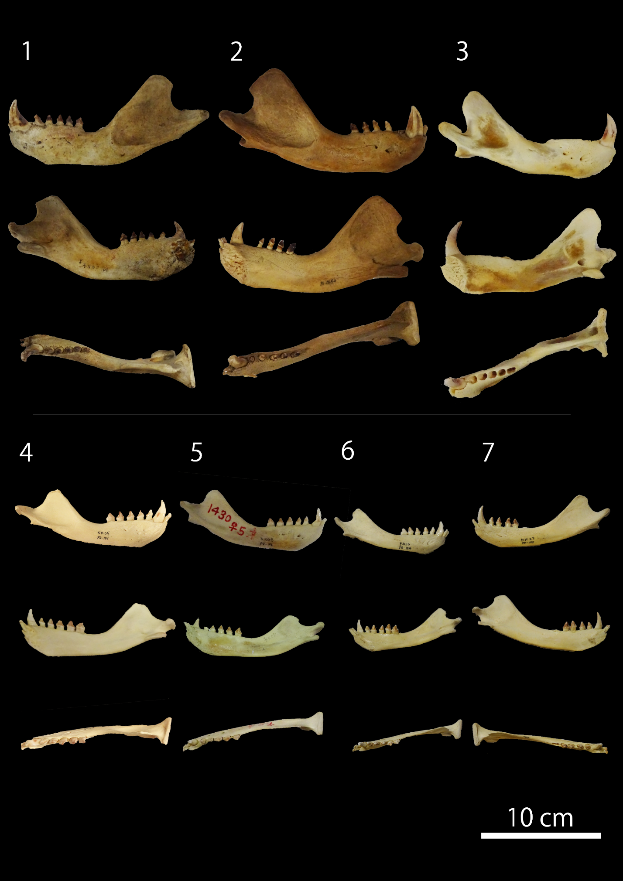
Plate 5

Plate 6

Specimens of female *Callorhinus ursinus* measured in this study. The upper row shows lateral aspect, the middle row shows medial aspect, and the lower row shows dorsal aspect, respectively. The scale shows 10 cm.

1：NMNS-KK 24

2：NMNS-KK 141

3：NMNS-KK 151

4：NMNS-M 1995

5：NMNS-M 35148

6：NMNS-M 42128


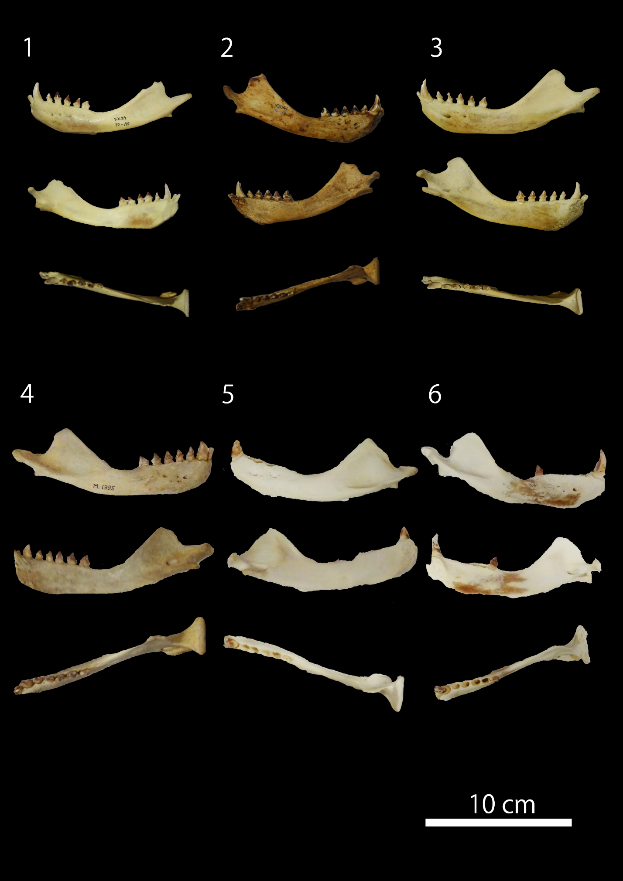
Plate 6

Plate 7

Specimens of male *Zalophus japonicus* measured in this study. The upper row shows lateral aspect, the middle row shows medial aspect, and the lower row shows dorsal aspect, respectively. The scale shows 10 cm.

1：HM-55953-18-1

2：HM-55953-18-2

3：HM-55953-18-3

4：HM-55953-18-4

5：HM-55953-18-5

6：HM-55953-18-6


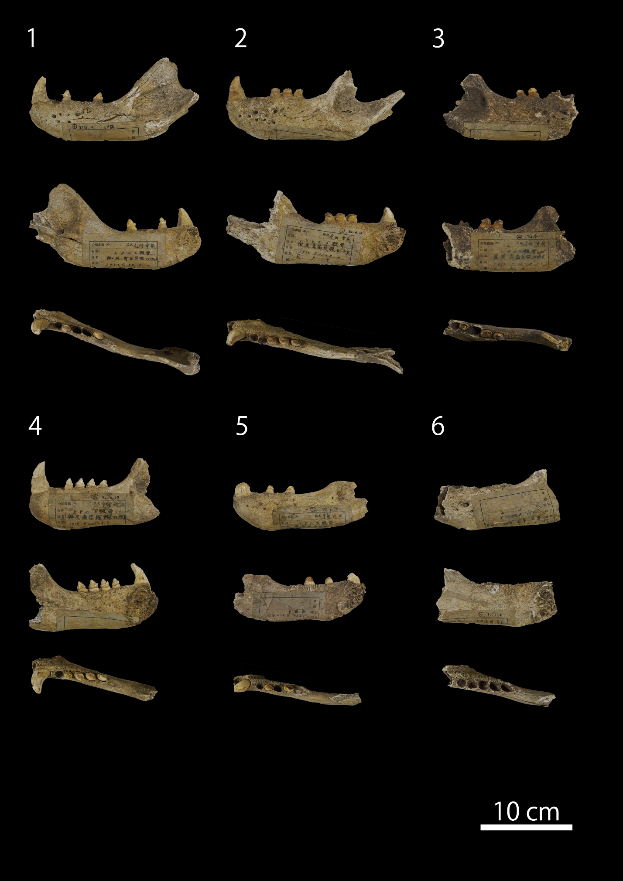
Plate 7

Plate 8

Specimens of *Zalophus japonicus* measured in this study. The upper row shows lateral aspect, the middle row shows medial aspect, and the lower row shows dorsal aspect, respectively. The scale shows 10 cm.

1：male, HM-55953-18-7

2：male, HM-55953-18-8

3：male, DCIFC-ER11H

4：male, DCIFC-HM2L

5：female, DCIFC-HM2・97R.No.30262


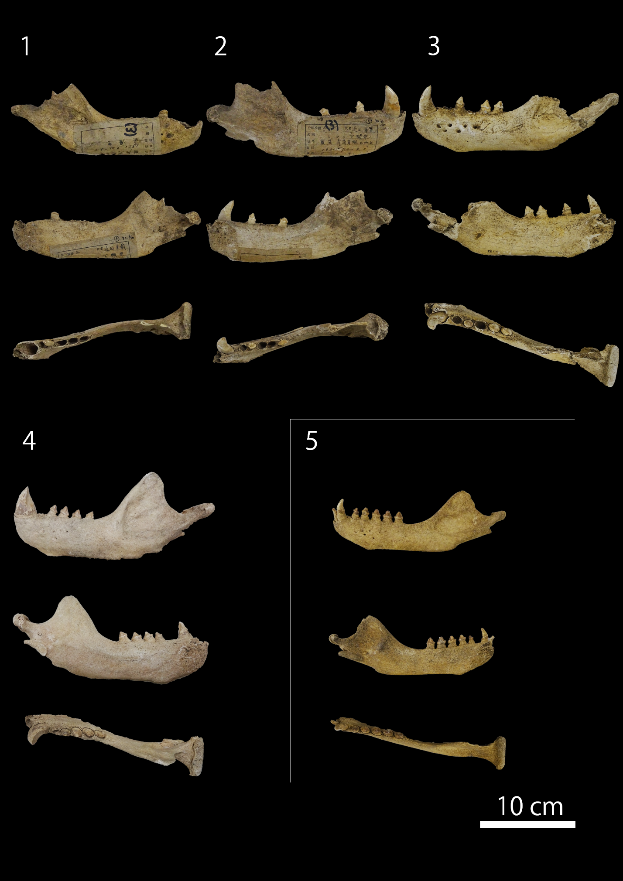
Plate 8
