# Supplementary material for: The oldest record of the Steller sea lion Eumetopias jubatus (Schreber, 1776) from the early Pleistocene of the North Pacific
Source: PeerJ. 2020 Aug 27;8:e9709. doi: 10.7717/peerj.9709 (PMC7456534; doi:10.7717/peerj.9709)
Supplement: Supplemental Information 3 — The numbers in parentheses correspond to each landmark. The raw data show all measurement results of mandibles of fur seals, sea lions, and the fossil. These data were used to identify the fossil. [file peerj-08-9709-s003.docx]

**Table S1.** Measurements (in mm) of mandibles for morphometric analyses

| ID | Registration number | A | B | C | D |
| --- | --- | --- | --- | --- | --- |
| *E. jubatus* [m]1 | NMNS-KK 14 | 262.00 | 232.00 | 62.39 | 31.12 |
| *E. jubatus* [m]2 | NMNS-KK 15 | 285.00 | 242.00 | 64.52 | 31.71 |
| *E. jubatus* [m]3 | NMNS-KK 23 | 265.80 | 242.20 | 53.06 | 30.16 |
| *E. jubatus* [m]4 | NMNS-KK 51 | 244.00 | 212.00 | 44.88 | 26.05 |
| *E. jubatus* [m]5 | NMNS-KK 63 | 237.00 | 208.98 | 39.38 | 26.09 |
| *E. jubatus* [m]6 | NMNS-KK 69 | 228.00 | 201.06 | 39.52 | 25.39 |
| *E. jubatus* [m]7 | NMNS-KK 73 | 227.00 | 202.19 | 48.39 | 24.85 |
| *E. jubatus* [m]8 | NMNS-KK 167 | 226.00 | 190.07 | 55.24 | 27.24 |
| *E. jubatus* [m]9 | NMNS-KK 169 | 282.50 | 257.20 | 49.62 | 31.37 |
| *E. jubatus* [m]10 | NMNS-KK 192 | 237.00 | 215.00 | 50.53 | 16.15 |
| *E. jubatus* [m]11 | NSMT-M 5627 | 284.00 | 265.00 | 53.57 | 37.20 |
| *E. jubatus* [m]12 | NSMT-M 28387 | 287.00 | 253.00 | 61.92 | 31.87 |
| *E. jubatus* [f]1 | NMNS-KK 11 | 200.71 | 181.51 | 28.23 | 16.15 |
| *E. jubatus* [f]2 | NMNS-KK 53 | 219.00 | 197.66 | 38.70 | 19.24 |
| *E. jubatus* [f]3 | NMNS-KK 54 | 214.00 | 194.95 | 29.08 | 16.67 |
| *E. jubatus* [f]4 | NMNS-KK 55 | 200.36 | 184.34 | 29.04 | 17.18 |
| *E. jubatus* [f]5 | NMNS-KK 56 | 200.26 | 185.80 | 29.31 | 16.33 |
| *E. jubatus* [f]6 | NMNS-KK 139 | 208.00 | 184.79 | 32.35 | 17.79 |
| *E. jubatus* [f]7 | NMNS-KK 146 | 215.00 | 193.55 | 35.94 | 18.80 |
| *E. jubatus* [f]8 | NMNS-KK 154 | 224.00 | 198.59 | 39.09 | 18.52 |
| *E. jubatus* [f]9 | NMNS-KK 158 | 222.00 | 202.09 | 36.87 | 19.31 |
| *E. jubatus* [f]10 | NMNS-KK 165 | 220.70 | 200.47 | 33.91 | 16.87 |
| *E. jubatus* [f]11 | NMNS-KK 166 | 224.00 | 207.23 | 31.49 | 16.14 |
| *E. jubatus* [f]12 | NSMT-M 17123 | 228.00 | 209.30 | 36.87 | 19.57 |
| *C. ursinus* [m]1 | NSMT-M 2454 | 165.14 | 153.65 | 27.64 | 18.40 |
| *C. ursinus* [m]2 | NSMT-M 17140 | 179.11 | 163.67 | 29.76 | 17.01 |
| *C. ursinus* [m]3 | NSMT-M 46874 | 146.15 | 136.19 | 22.06 | 17.69 |
| *C. ursinus* [f]1 | NMNS-KK 05 | 126.30 | 113.00 | 21.52 | 10.13 |
| *C. ursinus* [f]2 | NMNS-KK 08 | 120.12 | 110.57 | 19.38 | 10.58 |
| *C. ursinus* [f]3 | NMNS-KK 10 | 96.26 | 89.41 | 12.63 | 4.94 |
| *C. ursinus* [f]4 | NMNS-KK 22 | 118.07 | 110.00 | 14.29 | 9.79 |
| *C. ursinus* [f]5 | NMNS-KK 24 | 118.77 | 106.03 | 14.14 | 9.57 |
| *C. ursinus* [f]6 | NMNS-KK 141 | 114.34 | 107.35 | 13.63 | 9.63 |
| *C. ursinus* [f]7 | NMNS-KK 151 | 120.19 | 111.13 | 16.70 | 9.43 |
| *C. ursinus* [f]8 | NSMT-M 1995 | 128.72 | 120.21 | 18.92 | 8.65 |
| *C. ursinus* [f]9 | NSMT-M 35148 | 123.51 | 117.89 | 17.32 | 8.84 |
| *C. ursinus* [f]10 | NSMT-M 42128 | 130.01 | 119.00 | 20.02 | 11.45 |
| *Z. japonicus* [m]1 | HM-55953-18-1 | - | - | - | 22.91 |
| *Z. japonicus* [m]2 | HM-55953-18-2 | - | - | 34.92 | 22.02 |
| *Z. japonicus* [m]3 | HM-55953-18-3 | - | - | - | - |
| *Z. japonicus* [m]4 | HM-55953-18-4 | - | - | 34.59 | 23.45 |
| *Z. japonicus* [m]5 | HM-55953-18-5 | - | - | 33.18 | 18.14 |
| *Z. japonicus* [m]6 | HM-55953-18-6 | - | - | - | - |
| *Z. japonicus* [m]7 | HM-55953-18-7 | 204.72 | - | - | 21.49 |
| *Z. japonicus* [m]8 | HM-55953-18-8 | 225.00 | 203.61 | 42.45 | 25.46 |
| *Z. japonicus* [m]9 | DCIFC-ER11H | 226.00 | 211.00 | 35.77 | 21.51 |
| *Z. japonicus* [m]10 | DCIFC-HM2 | 234.50 | 204.48 | 42.08 | 25.25 |
| *Z. japonicus* [f]1 | DCIFC-HM2・97R.No.30262 | 183.66 | 173.44 | 23.73 | 14.00 |
| *P. ulysses* [m]1 | USNM 187109  (*Barnes et al., 2006*) | - | - | - | - |
| *P. ulysses* [m]2 | UCMP 219377  (*Poust & Boessenecker, 2017*) | - | - | - | - |
| GKZ-N 00001 | | - | - | 50.35 | 42.76 |

| ID | E | F | G | H | I | J | K |
| --- | --- | --- | --- | --- | --- | --- | --- |
| *E. jubatus* [m]1 | 63.12 | 59.01 | 43.99 | 46.83 | 62.56 | 62.65 | 57.82 |
| *E. jubatus* [m]2 | 70.48 | 75.58 | 44.33 | 62.52 | 63.13 | 63.50 | 64.56 |
| *E. jubatus* [m]3 | 70.53 | 60.28 | 47.98 | 50.82 | 61.99 | 63.89 | 63.62 |
| *E. jubatus* [m]4 | 55.94 | 52.04 | 38.46 | 39.89 | 51.77 | 55.91 | 45.21 |
| *E. jubatus* [m]5 | 51.21 | 49.44 | 37.25 | 37.93 | 45.60 | 49.02 | 41.41 |
| *E. jubatus* [m]6 | 51.19 | 48.07 | 36.39 | 37.22 | 62.98 | 49.68 | 43.61 |
| *E. jubatus* [m]7 | 48.46 | 49.25 | 39.05 | 39.12 | 48.61 | 52.58 | 43.64 |
| *E. jubatus* [m]8 | 48.99 | 34.04 | 30.51 | 29.41 | 50.34 | 50.11 | 41.85 |
| *E. jubatus* [m]9 | 72.71 | 45.21 | 40.08 | 49.14 | 63.85 | 66.30 | 67.95 |
| *E. jubatus* [m]10 | 50.37 | 46.99 | 38.65 | 37.59 | 50.20 | 54.06 | 44.05 |
| *E. jubatus* [m]11 | 61.91 | 68.54 | 48.99 | 51.93 | 52.38 | 71.69 | 65.89 |
| *E. jubatus* [m]12 | 73.00 | 53.00 | 48.07 | 48.10 | 58.54 | 70.32 | 66.82 |
| *E. jubatus* [f]1 | 41.56 | 31.21 | 22.36 | 29.35 | 32.11 | 33.06 | 32.51 |
| *E. jubatus* [f]2 | 46.49 | 30.97 | 23.70 | 26.57 | 38.76 | 40.71 | 38.70 |
| *E. jubatus* [f]3 | 45.99 | 33.75 | 21.97 | 25.95 | 36.04 | 36.31 | 36.07 |
| *E. jubatus* [f]4 | 41.91 | 35.98 | 25.63 | 31.20 | 32.47 | 36.02 | 33.61 |
| *E. jubatus* [f]5 | 46.88 | 33.47 | 24.22 | 24.61 | 36.09 | 39.52 | 36.29 |
| *E. jubatus* [f]6 | 42.95 | 33.14 | 24.11 | 26.95 | 32.51 | 32.68 | 37.71 |
| *E. jubatus* [f]7 | 42.68 | 34.11 | 24.75 | 33.18 | 34.47 | 36.35 | 35.17 |
| *E. jubatus* [f]8 | 44.16 | 33.43 | 25.47 | 25.94 | 38.71 | 40.28 | 37.99 |
| *E. jubatus* [f]9 | 45.38 | 38.43 | 28.72 | 34.17 | 37.13 | 40.25 | 38.88 |
| *E. jubatus* [f]10 | 45.15 | 40.36 | 30.70 | 30.89 | 42.74 | 41.65 | 41.27 |
| *E. jubatus* [f]11 | 49.35 | 32.56 | 27.39 | 26.83 | 36.98 | 40.93 | 42.56 |
| *E. jubatus* [f]12 | 49.16 | 29.28 | 25.76 | 26.03 | 33.42 | 41.89 | 41.22 |
| *C. ursinus* [m]1 | 41.43 | 36.82 | 26.26 | 33.41 | 23.33 | 34.37 | 30.79 |
| *C. ursinus* [m]2 | 41.16 | 30.29 | 25.66 | 28.71 | 27.39 | 34.80 | 33.70 |
| *C. ursinus* [m]3 | 43.60 | 34.59 | 27.78 | 28.24 | 25.43 | 32.18 | 30.31 |
| *C. ursinus* [f]1 | 24.68 | 20.45 | 17.53 | 16.03 | 18.68 | 21.94 | 18.95 |
| *C. ursinus* [f]2 | 21.26 | 16.92 | 14.82 | 15.78 | 17.04 | 17.05 | 17.56 |
| *C. ursinus* [f]3 | 17.91 | 15.68 | 13.25 | 13.90 | 15.04 | 13.83 | 13.54 |
| *C. ursinus* [f]4 | 20.44 | 21.48 | 17.55 | 17.02 | 14.05 | 16.62 | 15.82 |
| *C. ursinus* [f]5 | 22.15 | 17.83 | 15.67 | 18.34 | 15.61 | 16.54 | 16.93 |
| *C. ursinus* [f]6 | 21.94 | 17.52 | 16.52 | 15.46 | 14.38 | 15.68 | 16.01 |
| *C. ursinus* [f]7 | 20.97 | 15.82 | 15.69 | 12.49 | 16.35 | 16.33 | 17.63 |
| *C. ursinus* [f]8 | 24.46 | 16.01 | 11.78 | 13.41 | 16.39 | 20.29 | 17.24 |
| *C. ursinus* [f]9 | 23.86 | 15.39 | 12.67 | 11.11 | 15.44 | 18.00 | 18.85 |
| *C. ursinus* [f]10 | 31.57 | 24.20 | 21.58 | 20.52 | 16.82 | 20.01 | 19.29 |
| *Z. japonicus* [m]1 | - | - | 28.29 | 34.02 | 36.37 | 40.27 | 42.99 |
| *Z. japonicus* [m]2 | - | 41.93 | 26.35 | 35.23 | 35.53 | 41.40 | 49.47 |
| *Z. japonicus* [m]3 | - | - | - | - | - | 47.70 | 47.95 |
| *Z. japonicus* [m]4 | - | 35.79 | 34.96 | 36.79 | 36.23 | - | 45.05 |
| *Z. japonicus* [m]5 | - | 23.55 | 18.00 | 19.40 | 28.40 | 43.30 | 43.84 |
| *Z. japonicus* [m]6 | - | - | - | - | - | 45.49 | 47.13 |
| *Z. japonicus* [m]7 | - | - | - | - | - | 37.89 | 39.35 |
| *Z. japonicus* [m]8 | - | 43.09 | 31.17 | 37.24 | 39.75 | 44.60 | 43.14 |
| *Z. japonicus* [m]9 | 47.46 | 39.80 | 33.99 | 34.65 | 31.89 | 41.52 | 42.07 |
| *Z. japonicus* [m]10 | 52.99 | 47.18 | 34.03 | 45.19 | 35.93 | 44.81 | 45.51 |
| *Z. japonicus* [f]1 | 33.93 | 27.56 | 21.01 | 22.95 | 16.32 | 26.19 | 29.07 |
| *P. ulysses* [m]1 | - | - | - | - | - | - | 48.9 |
| *P. ulysses* [m]2 | - | - | - | - | - | - | 32.75 |
| GKZ-N 00001 | - | - | - | - | 75.15 | 76.46 | 66.34 |

| ID | L | M | N | O | P | Q | R |
| --- | --- | --- | --- | --- | --- | --- | --- |
| *E. jubatus* [m]1 | 87.09 | 24.33 | 88.59 | 85.49 | 222.00 | 73.79 | 76.91 |
| *E. jubatus* [m]2 | 97.46 | 14.71 | 108.81 | 87.19 | 233.00 | 77.95 | 83.65 |
| *E. jubatus* [m]3 | 99.17 | 18.36 | 95.43 | 95.89 | 223.80 | 73.23 | 71.03 |
| *E. jubatus* [m]4 | 85.05 | 19.80 | 76.15 | 45.19 | 199.66 | 63.81 | 49.81 |
| *E. jubatus* [m]5 | 83.41 | 24.12 | 73.43 | 41.05 | 197.87 | 64.90 | 55.50 |
| *E. jubatus* [m]6 | 76.00 | 17.20 | 65.54 | 57.47 | 180.22 | 58.88 | 51.61 |
| *E. jubatus* [m]7 | 81.20 | 18.94 | 68.30 | 54.13 | 185.41 | 56.53 | 50.29 |
| *E. jubatus* [m]8 | 75.34 | 14.60 | 66.69 | 61.68 | 186.53 | 53.42 | 58.08 |
| *E. jubatus* [m]9 | 104.17 | 17.66 | 105.13 | 87.31 | 229.10 | 86.02 | 83.74 |
| *E. jubatus* [m]10 | 80.81 | 19.97 | 71.33 | 58.69 | 187.42 | 69.19 | 61.20 |
| *E. jubatus* [m]11 | 103.86 | 28.60 | 100.56 | 78.59 | 237.00 | 85.34 | 77.67 |
| *E. jubatus* [m]12 | 108.34 | 17.35 | 96.82 | 83.75 | 202.00 | 73.16 | 70.96 |
| *E. jubatus* [f]1 | 75.82 | 18.53 | 59.87 | 57.05 | 170.32 | 53.31 | 54.23 |
| *E. jubatus* [f]2 | 63.31 | 35.32 | 67.04 | 61.09 | 172.56 | 53.69 | 52.75 |
| *E. jubatus* [f]3 | 68.28 | 24.49 | 59.97 | 51.86 | 180.47 | 50.44 | 41.83 |
| *E. jubatus* [f]4 | 74.03 | 15.51 | 60.50 | 59.31 | 163.64 | 56.54 | 48.94 |
| *E. jubatus* [f]5 | 73.18 | 19.59 | 63.62 | 61.82 | 169.45 | 45.92 | 41.86 |
| *E. jubatus* [f]6 | 74.47 | 18.68 | 63.44 | 64.66 | 167.85 | 50.25 | 44.03 |
| *E. jubatus* [f]7 | 73.34 | 21.02 | 63.08 | 61.39 | 175.74 | 55.17 | 50.95 |
| *E. jubatus* [f]8 | 75.67 | 21.69 | 68.08 | 60.48 | 177.65 | 59.19 | 52.51 |
| *E. jubatus* [f]9 | 77.43 | 23.63 | 67.23 | 61.21 | 170.80 | 62.97 | 49.88 |
| *E. jubatus* [f]10 | 78.71 | 19.08 | 60.69 | 61.16 | 177.66 | 55.39 | 53.47 |
| *E. jubatus* [f]11 | 82.89 | 19.94 | 66.34 | 65.21 | 184.31 | 59.18 | 63.50 |
| *E. jubatus* [f]12 | 82.39 | 20.90 | 72.06 | 56.62 | 178.60 | 59.88 | 59.53 |
| *C. ursinus* [m]1 | 50.27 | 19.92 | 60.90 | 46.80 | 135.84 | 52.65 | 41.72 |
| *C. ursinus* [m]2 | 48.88 | 23.34 | 74.72 | 50.02 | 148.30 | 68.00 | 62.03 |
| *C. ursinus* [m]3 | 42.98 | 25.33 | 57.99 | 35.98 | 138.29 | 48.62 | 48.48 |
| *C. ursinus* [f]1 | 36.68 | 17.42 | 35.14 | 31.27 | 97.12 | 39.70 | 27.04 |
| *C. ursinus* [f]2 | 37.54 | 17.69 | 36.22 | 31.71 | 93.95 | 32.68 | 28.89 |
| *C. ursinus* [f]3 | 30.79 | 14.44 | 24.93 | 24.03 | 80.96 | 23.49 | 15.17 |
| *C. ursinus* [f]4 | 37.05 | 15.20 | 33.98 | 31.43 | 95.34 | 31.52 | 20.53 |
| *C. ursinus* [f]5 | 36.16 | 17.27 | - | - | - | - | 24.51 |
| *C. ursinus* [f]6 | 37.87 | 15.78 | 33.23 | 36.06 | 91.47 | 32.24 | 19.71 |
| *C. ursinus* [f]7 | 38.97 | 14.46 | 32.98 | 32.50 | 96.01 | 32.27 | 17.99 |
| *C. ursinus* [f]8 | 37.09 | 17.96 | 35.59 | 29.66 | 98.57 | 38.91 | 33.02 |
| *C. ursinus* [f]9 | 37.90 | 18.86 | 37.11 | 31.58 | 96.93 | 37.08 | 35.09 |
| *C. ursinus* [f]10 | 38.91 | 21.48 | 36.90 | 35.17 | 101.58 | 40.84 | 36.45 |
| *Z. japonicus* [m]1 | 72.44 | 20.15 | 82.21 | 73.75 | - | - | 43.66 |
| *Z. japonicus* [m]2 | 73.41 | 27.76 | - | - | - | - | - |
| *Z. japonicus* [m]3 | - | 25.80 | - | - | - | - | - |
| *Z. japonicus* [m]4 | 76.90 | 23.21 | - | - | - | - | - |
| *Z. japonicus* [m]5 | 71.54 | 23.05 | - | - | - | - | - |
| *Z. japonicus* [m]6 | - | 17.29 | - | - | - | - | - |
| *Z. japonicus* [m]7 | - | 21.63 | - | - | - | - | 52.93 |
| *Z. japonicus* [m]8 | 71.02 | 23.91 | - | - | - | - | 59.54 |
| *Z. japonicus* [m]9 | 83.02 | 22.25 | - | - | - | - | - |
| *Z. japonicus* [m]10 | 69.12 | 22.60 | 96.36 | 62.78 | 182.04 | 78.42 | 51.58 |
| *Z. japonicus* [f]1 | 64.26 | 23.88 | 56.79 | 45.28 | 143.82 | 51.91 | 34.99 |
| *P. ulysses* [m]1 | - | - | - | - | - | - | - |
| *P. ulysses* [m]2 | - | - | - | - | - | - | - |
| GKZ-N 00001 | 100.13 | 27.60 | - | - | - | - | - |

| ID | S | T | U | V | W | X | Y |
| --- | --- | --- | --- | --- | --- | --- | --- |
| *E. jubatus* [m]1 | 57.82 | 13.73 | 25.05 | 48.64 | 28.40 | 65.63 | 47.96 |
| *E. jubatus* [m]2 | 64.28 | 16.37 | 34.93 | 53.21 | 28.24 | 73.08 | 51.58 |
| *E. jubatus* [m]3 | 58.48 | 15.79 | 26.59 | 44.43 | 30.35 | 72.94 | 46.82 |
| *E. jubatus* [m]4 | 43.19 | 6.07 | 21.94 | 37.20 | 12.80 | 63.53 | 41.33 |
| *E. jubatus* [m]5 | 50.30 | 7.28 | 33.47 | 29.34 | 10.44 | 56.08 | 38.82 |
| *E. jubatus* [m]6 | 40.31 | 8.43 | 20.48 | 32.96 | 10.90 | 54.24 | 35.75 |
| *E. jubatus* [m]7 | 37.38 | 7.71 | 18.62 | 29.13 | 12.69 | 57.46 | 35.70 |
| *E. jubatus* [m]8 | 38.93 | 10.74 | 23.65 | 40.01 | 23.36 | 54.18 | 32.71 |
| *E. jubatus* [m]9 | 60.22 | 16.37 | 30.09 | 44.67 | 26.82 | 76.41 | 49.71 |
| *E. jubatus* [m]10 | 43.97 | 7.20 | 29.59 | 31.38 | 14.20 | 58.50 | 38.84 |
| *E. jubatus* [m]11 | 56.12 | 11.86 | 29.83 | 34.97 | 33.59 | 80.05 | 46.61 |
| *E. jubatus* [m]12 | 45.60 | 14.54 | 24.40 | 26.77 | 35.85 | 72.84 | 44.18 |
| *E. jubatus* [f]1 | 32.64 | 15.08 | 26.50 | 14.96 | 23.18 | 41.07 | 23.69 |
| *E. jubatus* [f]2 | 37.39 | 8.55 | 22.27 | 29.62 | 16.71 | 42.72 | 28.65 |
| *E. jubatus* [f]3 | 38.52 | 6.09 | 18.36 | 29.16 | 9.59 | 47.46 | 28.71 |
| *E. jubatus* [f]4 | 45.10 | 4.60 | 24.51 | 37.28 | 8.58 | 42.97 | 24.85 |
| *E. jubatus* [f]5 | 33.37 | 6.82 | 19.36 | 34.46 | 8.57 | 44.24 | 29.54 |
| *E. jubatus* [f]6 | 33.96 | 10.89 | 15.54 | 24.15 | 18.18 | 37.25 | 24.54 |
| *E. jubatus* [f]7 | 41.12 | 9.23 | 22.00 | 35.86 | 16.93 | 41.21 | 28.56 |
| *E. jubatus* [f]8 | 38.30 | 15.94 | 21.06 | 32.51 | 20.02 | 44.53 | 30.45 |
| *E. jubatus* [f]9 | 0.34 | 12.63 | 26.21 | 25.94 | 20.52 | 46.72 | 28.04 |
| *E. jubatus* [f]10 | 43.71 | 7.58 | 17.67 | 31.56 | 12.17 | 49.15 | 29.11 |
| *E. jubatus* [f]11 | 48.81 | 7.40 | 26.47 | 41.15 | 8.72 | 52.17 | 31.09 |
| *E. jubatus* [f]12 | 47.84 | 7.97 | 31.76 | 44.49 | 14.74 | 47.08 | 29.38 |
| *C. ursinus* [m]1 | 29.53 | 14.80 | 20.93 | 17.99 | 17.35 | 35.12 | 24.59 |
| *C. ursinus* [m]2 | 49.05 | 14.96 | 32.26 | 32.77 | 22.25 | 38.42 | 21.61 |
| *C. ursinus* [m]3 | 30.24 | 19.95 | 21.35 | 20.08 | 30.83 | 36.13 | 21.79 |
| *C. ursinus* [f]1 | 17.08 | 8.48 | 8.94 | 6.89 | 12.90 | 23.83 | 11.68 |
| *C. ursinus* [f]2 | 16.59 | 5.33 | 11.84 | 12.14 | 7.69 | 23.07 | 10.65 |
| *C. ursinus* [f]3 | 11.17 | 3.51 | 6.56 | 9.65 | 5.39 | 16.25 | 8.61 |
| *C. ursinus* [f]4 | 13.63 | 2.30 | 8.82 | 8.79 | 4.66 | 19.09 | 10.31 |
| *C. ursinus* [f]5 | 18.59 | 8.77 | - | - | 6.76 | 17.34 | 11.70 |
| *C. ursinus* [f]6 | 13.48 | 5.63 | 8.75 | 8.97 | 6.47 | 18.84 | 10.51 |
| *C. ursinus* [f]7 | 15.14 | 2.21 | 10.58 | 9.61 | 3.44 | 21.57 | 10.76 |
| *C. ursinus* [f]8 | 15.63 | 11.35 | 8.74 | 9.00 | 17.63 | 23.97 | 12.20 |
| *C. ursinus* [f]9 | 21.89 | 8.34 | 16.19 | 17.49 | 12.40 | 21.04 | 10.93 |
| *C. ursinus* [f]10 | 26.66 | 8.12 | 19.71 | 15.65 | 11.81 | 24.60 | 14.68 |
| *Z. japonicus* [m]1 | 34.22 | 9.93 | 18.02 | 24.78 | 14.44 | 45.85 | 30.05 |
| *Z. japonicus* [m]2 | - | - | - | - | - | 44.80 | 30.69 |
| *Z. japonicus* [m]3 | - | - | - | - | - | - | - |
| *Z. japonicus* [m]4 | - | - | - | - | - | - | - |
| *Z. japonicus* [m]5 | - | - | - | - | - | 49.37 | 28.62 |
| *Z. japonicus* [m]6 | - | - | - | - | - | - | 35.13 |
| *Z. japonicus* [m]7 | - | - | - | - | - | 49.04 | 28.33 |
| *Z. japonicus* [m]8 | - | - | - | - | - | 51.94 | 33.25 |
| *Z. japonicus* [m]9 | - | - | - | - | - | 48.92 | 31.99 |
| *Z. japonicus* [m]10 | 32.11 | 22.32 | 21.63 | 21.55 | 19.01 | 49.76 | 31.24 |
| *Z. japonicus* [f]1 | 32.27 | 4.80 | 19.46 | 22.59 | 10.30 | 37.79 | 18.67 |
| *P. ulysses* [m]1 | - | - | - | - | - | 56 | 36 |
| *P. ulysses* [m]2 | - | - | - | - | - | 38.8 | 11.8 |
| GKZ-N 00001 | - | - | - | - | - | 81.31 | 50.29 |

| ID | Z | AA | AB | AC | AD | AE | AF |
| --- | --- | --- | --- | --- | --- | --- | --- |
| *E. jubatus* [m]1 | 79.09 | 55.51 | 8.89 | 112.38 | 69.32 | 17.92 | 27.70 |
| *E. jubatus* [m]2 | 68.22 | 60.37 | 10.92 | 122.34 | 76.88 | 18.60 | 28.91 |
| *E. jubatus* [m]3 | 84.57 | 63.67 | 11.04 | 118.02 | 74.37 | 21.37 | 31.61 |
| *E. jubatus* [m]4 | 70.24 | 42.35 | 7.04 | 108.30 | 69.13 | 16.81 | 26.84 |
| *E. jubatus* [m]5 | 63.42 | 44.04 | 7.00 | 104.39 | 70.63 | 17.55 | 25.27 |
| *E. jubatus* [m]6 | 55.26 | 37.11 | 7.48 | 104.15 | 67.12 | 16.16 | 25.46 |
| *E. jubatus* [m]7 | 57.83 | 40.50 | 6.87 | 99.00 | 64.76 | 15.66 | 23.19 |
| *E. jubatus* [m]8 | 45.74 | 38.69 | 8.54 | 99.07 | 64.23 | 16.25 | 24.31 |
| *E. jubatus* [m]9 | 95.29 | 50.63 | 11.83 | 117.86 | 68.21 | 19.62 | 30.97 |
| *E. jubatus* [m]10 | 59.04 | 40.29 | 8.16 | 103.34 | 63.31 | 16.05 | 25.44 |
| *E. jubatus* [m]11 | 72.65 | 53.41 | 11.79 | 122.41 | 76.82 | 22.18 | 33.72 |
| *E. jubatus* [m]12 | 68.05 | 44.78 | 12.17 | 121.08 | 78.21 | 18.26 | 32.83 |
| *E. jubatus* [f]1 | 49.30 | 37.10 | 7.57 | 93.96 | 68.26 | 8.61 | 12.59 |
| *E. jubatus* [f]2 | 53.38 | 36.25 | 6.46 | 91.90 | 62.69 | 11.15 | 17.54 |
| *E. jubatus* [f]3 | 65.96 | 36.37 | 6.65 | 89.41 | 62.22 | 8.99 | 14.92 |
| *E. jubatus* [f]4 | 57.09 | 35.49 | 6.77 | 86.70 | 61.27 | 9.11 | 15.77 |
| *E. jubatus* [f]5 | 62.13 | 39.75 | 7.75 | 86.59 | 62.26 | 8.58 | 15.41 |
| *E. jubatus* [f]6 | 52.15 | 34.48 | 5.74 | 92.33 | 63.71 | 10.07 | 13.42 |
| *E. jubatus* [f]7 | 52.28 | 35.45 | 9.66 | 93.11 | 65.32 | 9.26 | 13.55 |
| *E. jubatus* [f]8 | 57.31 | 39.28 | 7.48 | 91.46 | 67.80 | 10.15 | 15.33 |
| *E. jubatus* [f]9 | 62.77 | 38.90 | 8.45 | 93.88 | 66.63 | 9.83 | 15.97 |
| *E. jubatus* [f]10 | 64.06 | 39.25 | 8.99 | 94.27 | 66.60 | 10.50 | 16.73 |
| *E. jubatus* [f]11 | 66.27 | 39.73 | 8.69 | 96.19 | 68.77 | 10.99 | 15.90 |
| *E. jubatus* [f]12 | 58.27 | 42.35 | 7.13 | 91.59 | 68.44 | 11.22 | 17.41 |
| *C. ursinus* [m]1 | 51.12 | 31.61 | 6.22 | 64.81 | 43.82 | 10.54 | 17.06 |
| *C. ursinus* [m]2 | 55.39 | 33.32 | 7.87 | 66.49 | 44.87 | 11.06 | 15.32 |
| *C. ursinus* [m]3 | 44.65 | 24.97 | 5.22 | 62.12 | 56.85 | 10.49 | 14.59 |
| *C. ursinus* [f]1 | 27.22 | 17.91 | 5.45 | 50.92 | 39.07 | 6.05 | 7.42 |
| *C. ursinus* [f]2 | 28.47 | 17.47 | 4.06 | 47.27 | 33.39 | 4.91 | 7.58 |
| *C. ursinus* [f]3 | 21.63 | 12.16 | 3.04 | 40.01 | 28.89 | 4.63 | 7.24 |
| *C. ursinus* [f]4 | 30.17 | 16.73 | 2.07 | 46.68 | 34.13 | 5.69 | 6.72 |
| *C. ursinus* [f]5 | 31.45 | 19.24 | 2.83 | 46.06 | 32.84 | 5.27 | 7.58 |
| *C. ursinus* [f]6 | 27.62 | 15.26 | 2.43 | 44.27 | 33.15 | 5.17 | 7.85 |
| *C. ursinus* [f]7 | 30.39 | 18.58 | 3.22 | 47.28 | 34.34 | 5.28 | 7.91 |
| *C. ursinus* [f]8 | 31.11 | 16.48 | 4.29 | 48.69 | 36.13 | 5.21 | 7.27 |
| *C. ursinus* [f]9 | 29.42 | 19.45 | 3.85 | 46.68 | 36.82 | 7.97 | 9.16 |
| *C. ursinus* [f]10 | 30.44 | 17.97 | 4.15 | 52.68 | 47.80 | 10.07 | 9.72 |
| *Z. japonicus* [m]1 | 59.92 | 35.33 | 10.55 | 86.43 | 55.85 | 13.16 | 20.59 |
| *Z. japonicus* [m]2 | 60.73 | 38.25 | 9.96 | 90.92 | 60.09 | 13.86 | 20.33 |
| *Z. japonicus* [m]3 | - | 34.16 | 11.15 | - | 61.34 | - | - |
| *Z. japonicus* [m]4 | - | - | - | 91.56 | 58.95 | 15.77 | 24.25 |
| *Z. japonicus* [m]5 | - | 29.91 | 13.27 | 88.42 | 60.50 | 13.52 | 22.62 |
| *Z. japonicus* [m]6 | - | - | - | - | 63.56 | - | - |
| *Z. japonicus* [m]7 | 46.65 | 28.79 | 8.48 | 87.63 | 57.54 | 13.43 | 23.68 |
| *Z. japonicus* [m]8 | 52.35 | 31.42 | 9.93 | 91.86 | 59.25 | 13.72 | 20.08 |
| *Z. japonicus* [m]9 | - | 37.61 | 8.13 | 95.60 | 66.04 | 12.59 | 22.73 |
| *Z. japonicus* [m]10 | 64.57 | 52.62 | 11.09 | 100.06 | 66.43 | 14.84 | 24.64 |
| *Z. japonicus* [f]1 | 39.03 | 26.62 | 6.67 | 74.52 | 56.84 | 9.74 | 11.77 |
| *P. ulysses* [m]1 | - | - | - | - | 64.8 | 17.2 | 26.8 |
| *P. ulysses* [m]2 | - | - | - | - | 57.6 | 13.7 | 20.4 |
| GKZ-N 00001 | 68.43 | 45.73 | 13.18 | 123.89 | 84.03 | 27.32 | 35.00 |

| ID | AG | AH | AI | AJ | AK | AL |
| --- | --- | --- | --- | --- | --- | --- |
| *E. jubatus* [m]1 | 9.78 | 11.62 | 12.69 | 12.45 | 11.78 | 7.41 |
| *E. jubatus* [m]2 | 9.34 | 11.43 | 14.57 | 13.18 | 9.96 | 6.94 |
| *E. jubatus* [m]3 | 11.72 | 13.41 | 13.91 | 12.93 | 12.50 | 7.93 |
| *E. jubatus* [m]4 | 8.66 | 12.83 | 14.50 | 13.03 | 10.32 | 4.54 |
| *E. jubatus* [m]5 | 11.22 | 13.01 | 14.72 | 12.51 | 11.60 | 5.96 |
| *E. jubatus* [m]6 | 9.24 | 11.79 | 10.50 | 13.08 | 13.13 | 3.88 |
| *E. jubatus* [m]7 | 11.38 | 13.23 | 14.04 | 12.03 | 9.73 | 3.39 |
| *E. jubatus* [m]8 | 8.36 | 10.06 | 10.94 | 11.08 | 8.84 | 3.72 |
| *E. jubatus* [m]9 | 8.48 | 12.58 | 14.78 | 13.32 | 12.65 | 12.39 |
| *E. jubatus* [m]10 | 9.98 | 12.81 | 15.09 | 13.11 | 10.79 | 4.37 |
| *E. jubatus* [m]11 | 12.10 | 13.30 | 15.49 | 15.76 | 12.81 | 11.59 |
| *E. jubatus* [m]12 | 13.44 | 14.28 | 16.35 | 15.99 | 12.94 | 4.90 |
| *E. jubatus* [f]1 | 7.52 | 8.83 | 10.37 | 10.34 | 11.46 | 3.94 |
| *E. jubatus* [f]2 | 9.56 | 10.73 | 12.21 | 10.76 | 8.62 | 3.13 |
| *E. jubatus* [f]3 | 7.91 | 9.22 | 11.65 | 10.61 | 8.44 | 5.14 |
| *E. jubatus* [f]4 | 8.78 | 9.71 | 10.65 | 9.68 | 9.73 | 3.31 |
| *E. jubatus* [f]5 | 8.63 | 10.31 | 12.50 | 11.49 | 10.12 | 4.62 |
| *E. jubatus* [f]6 | 8.68 | 9.68 | 11.31 | 10.23 | 9.53 | 4.81 |
| *E. jubatus* [f]7 | 7.60 | 9.14 | 11.34 | 10.01 | 9.71 | 5.17 |
| *E. jubatus* [f]8 | 9.89 | 9.67 | 11.72 | 9.19 | 7.47 | 4.39 |
| *E. jubatus* [f]9 | 8.21 | 10.51 | 12.19 | 10.80 | 9.21 | 3.73 |
| *E. jubatus* [f]10 | 8.30 | 12.41 | 12.37 | 11.59 | 9.64 | 4.04 |
| *E. jubatus* [f]11 | 9.21 | 11.01 | 13.38 | 13.18 | 11.16 | 4.54 |
| *E. jubatus* [f]12 | 8.12 | 10.05 | 11.94 | 10.92 | 10.35 | 6.28 |
| *C. ursinus* [m]1 | 7.31 | 7.27 | 7.36 | 7.24 | 8.32 | 1.65 |
| *C. ursinus* [m]2 | 4.45 | 5.43 | 5.72 | 5.48 | 6.72 | 2.24 |
| *C. ursinus* [m]3 | 8.31 | 8.11 | 7.57 | 7.44 | 7.94 | 1.78 |
| *C. ursinus* [f]1 | 5.59 | 6.31 | 7.25 | 7.46 | 7.79 | 0.00 |
| *C. ursinus* [f]2 | 4.26 | 3.87 | 5.22 | 5.06 | 5.48 | 1.92 |
| *C. ursinus* [f]3 | 3.58 | 4.71 | 4.64 | 4.61 | 5.76 | 0.00 |
| *C. ursinus* [f]4 | 4.04 | 4.59 | 4.71 | 4.91 | 5.88 | 0.89 |
| *C. ursinus* [f]5 | 4.62 | 5.29 | 5.24 | 4.83 | 5.74 | 1.07 |
| *C. ursinus* [f]6 | 4.24 | 5.67 | 5.11 | 4.72 | 5.33 | 1.71 |
| *C. ursinus* [f]7 | 4.60 | 4.55 | 5.05 | 4.85 | 5.48 | 2.93 |
| *C. ursinus* [f]8 | 4.70 | 5.28 | 5.70 | 5.85 | 7.45 | 0.00 |
| *C. ursinus* [f]9 | 7.43 | 7.38 | 7.38 | 6.75 | 7.58 | 0.00 |
| *C. ursinus* [f]10 | 7.00 | 6.89 | 7.46 | 7.50 | 5.79 | 1.57 |
| *Z. japonicus* [m]1 | 7.98 | 10.34 | 11.94 | 12.33 | 13.12 | 4.67 |
| *Z. japonicus* [m]2 | 9.69 | 12.58 | 12.49 | 14.32 | 12.11 | 6.41 |
| *Z. japonicus* [m]3 | 10.85 | 11.58 | 13.71 | 13.44 | 12.57 | 3.69 |
| *Z. japonicus* [m]4 | 10.16 | - | - | - | - | - |
| *Z. japonicus* [m]5 | 9.85 | 10.29 | 13.63 | 12.76 | 12.35 | 6.93 |
| *Z. japonicus* [m]6 | 11.55 | 12.44 | 13.18 | 12.69 | 13.90 | 5.51 |
| *Z. japonicus* [m]7 | 8.00 | 10.94 | 11.71 | 12.05 | 10.68 | 2.58 |
| *Z. japonicus* [m]8 | 8.38 | 11.72 | 10.97 | 13.99 | 11.53 | 7.10 |
| *Z. japonicus* [m]9 | 11.99 | 11.64 | 12.99 | 13.61 | 13.06 | 4.56 |
| *Z. japonicus* [m]10 | 9.53 | 12.46 | 13.60 | 15.02 | 14.16 | 4.32 |
| *Z. japonicus* [f]1 | 8.80 | 9.81 | 11.32 | 11.52 | 10.70 | 4.43 |
| *P. ulysses* [m]1 | - | - | - | - | - | - |
| *P. ulysses* [m]2 | - | - | - | - | - | - |
| GKZ-N 00001 | 13.33 | 12.79 | 16.07 | 14.51 | 18.42 | 10.04 |

**A**: total length (1-2)

**B**: caudal-most point of mandibular condyle－lateral edge of canine midpoint (2-3)

**C**: rostral tip of mandible－ventral edge of mandible underneath the canine (1-5)

**D**: width of mandible (lateral edge of canine midpoint－medial edge of mandibular symphysis caudal to the first incisor) (3-4)

**E**: length of mandibular condyle (10-11)

**F**: rostral tip of mandible－dorsal tip of canine (1-19)

**G**: lateral edge of canine midpoint－dorsal tip of canine (3-19)

**H**: medial edge of mandibular symphysis caudal to the first incisor－dorsal tip of canine (4-19)

**I**: depth of ramus at c1 (5-21)　　**J**: depth of ramus at p1 (8-9)　　**K**: depth of ramus at m1 (6-7)

**L**: ventral edge of mandible underneath the canine－ventral edge of mandible underneath the last postcanine (5-7)

**M**: lateral and caudal edge of last postcanine－rostral start point of the coronoid process (6-16)

**N**: depth of coronoid process (12-13)

**O**: ventral edge of mandible underneath the tip of the coronoid process－ventral edge of mandible underneath the start of the coronoid process (13-20)

**P**: rostral tip of mandible－dorsal-most point of coronoid process (1-12)

**Q**: caudal-most point of mandibular condyle－dorsal-most point of coronoid process (2-12)

**R**: width of coronoid process (14-15)　　　**S**: width of curving edge of coronoid process (17-18)

**T**: caudal-most point of coronoid process－caudal-most point on curving edge of coronoid process (14-18)

**U**: dorsal-most point of coronoid process－caudal-most point on curving edge of coronoid process (12-18)

**V**: dorsal-most point of coronoid process－rostral-most point on curving edge of coronoid process (12-17)

**W**: rostral-most point of coronoid process－rostral-most point on curving edge of coronoid process (15-17)

**X**: major axis of mandibular symphysis　　　**Y**: minor axis of mandibular symphysis

**Z**: major axis of masseteric fossa　　　**AA**: minor axis of masseteric fossa

**AB**: depth of masseteric fossa

**AC**: tooth length　　　**AD**: cheek tooth length　　　**AE**: transverse width of c1

**AF**: mesiodistal diameter of c1　　　**AG**: anteroposterior length of p1

**AH**: anteroposterior length of p2 **AI**: anteroposterior length of p3

**AJ**: anteroposterior length of p4　　　**AK**: anteroposterior length of m1

**AL**: diastema length between c1 and p1
